# Biomechanical properties of bone in a mouse model of Rett syndrome

**DOI:** 10.1016/j.bone.2014.10.008

**Published:** 2015-02

**Authors:** Bushra Kamal, David Russell, Anthony Payne, Diogo Constante, K. Elizabeth Tanner, Hanna Isaksson, Neashan Mathavan, Stuart R. Cobb

**Affiliations:** a Institute of Neuroscience and Psychology, University of Glasgow, Glasgow G12 8QQ, UK; b Laboratory of Human Anatomy, School of Life Sciences, University of Glasgow, Glasgow G12 8QQ, UK; c Department of Anatomy, Khyber Medical University, Khyber Pakhtunkhwa, Pakistan; d School of Engineering, University of Glasgow, Glasgow G12 8QQ, UK; e Faculadade de Engenharia da Universidade do Porto, Porto, Portugal; f Department of Orthopaedics, Clinical Sciences, Lund University, Lund, S-22185, Sweden; g Department of Biomedical Engineering, Lund University, Lund, Sweden

**Keywords:** Rett syndrome, MEPC2, Biomechanical properties, Cortical bone, Cancellous bone

## Abstract

Rett syndrome (RTT) is an X-linked genetic disorder and a major cause of intellectual disability in girls. Mutations in the methyl-CpG binding protein 2 (*MECP2*) gene are the primary cause of the disorder. Despite the dominant neurological phenotypes, *MECP2* is expressed ubiquitously throughout the body and a number of peripheral phenotypes such as scoliosis, reduced bone mineral density and skeletal fractures are also common and important clinical features of the disorder. In order to explore whether MeCP2 protein deficiency results in altered structural and functional properties of bone and to test the potential reversibility of any defects, we have conducted a series of histological, imaging and biomechanical tests of bone in a functional knockout mouse model of RTT. Both hemizygous *Mecp2*^stop/y^ male mice in which *Mecp2* is silenced in all cells and female *Mecp2*^stop/+^ mice in which *Mecp2* is silenced in ~ 50% of cells as a consequence of random X-chromosome inactivation, revealed significant reductions in cortical bone stiffness, microhardness and tensile modulus. Microstructural analysis also revealed alterations in both cortical and cancellous femoral bone between wild-type and MeCP2-deficient mice. Furthermore, unsilencing of *Mecp2* in adult mice cre-mediated stop cassette deletion resulted in a restoration of biomechanical properties (stiffness, microhardness) towards wild-type levels. These results show that MeCP2-deficiency results in overt, but potentially reversible, alterations in the biomechanical integrity of bone and highlights the importance of targeting skeletal phenotypes in considering the development of pharmacological and gene-based therapies.

## Highlights

• Functional *Mecp2* knockout mice display a range of deficits in skeletal properties including mechanical strength, stiffness and microhardness.
• Biomechanical deficits were accompanied by microstructural changes such as decreased trabecular thickness.
• Defects in biomechanical and material properties were ameliorated by unsilencing the *Mecp2* gene.

## Introduction

Rett syndrome (RTT), traditionally considered a neurodevelopmental disorder, mainly affects girls and is due principally to mutations in the X-linked gene methyl-CpG-binding protein 2 (*MECP2*) [1,2]. The age of onset is typically around 6–18 months after birth with characteristic symptoms including loss of speech, reduced head growth, stereotypic hand movements, motor dysfunction and autism-like features [2]. Whilst it is well established that the majority (> 95%) of classical RTT cases are due to mutations in the *MECP2* gene, the underlying function and regulation of MeCP2 protein remains unclear [3–6]. MeCP2 is a nuclear protein and is especially abundant in the brain. However, it is also expressed throughout the body [7–9] and in addition to the neurological phenotypes, a number of overt peripheral phenotypes are also common in RTT. For instance, spinal deformity (principally scoliosis and excessive kyphosis) is a very common feature, with ~ 50–90% of patients developing severe scoliosis [10–12], many of whom require corrective surgery. Other prominent skeletal anomalies include early osteoporosis, osteopenia, bone fractures and hip deformities [13–17]. Previous studies have found that Rett syndrome patients have reduced bone mass [18–21]. As a result, RTT patients have an increased risk of fractures and commonly sustain low-energy fractures [22]. Whilst MeCP2 is known to be expressed in bone tissues and studies have suggested a role of the protein in osteoclastogenesis [23], the role of MeCP2 in bone homeostasis is poorly defined.

The monogenic nature of RTT enables the disorder to be modelled in experimental animals. Many lines of mice have been developed in which *Mecp2* has been deleted, silenced or mutated to mimic major human mutations. These mouse lines replicate many of the features observed in RTT patients [5,24–28] and provide valuable tools for investigating MeCP2-related function/dysfunctions. An initial investigation into the skeletal system in *Mecp2*-knockout mice revealed a range of skeletal phenotypes including alterations in skeletal size, growth plate abnormalities and alternations in cortical and trabecular bone mass and mineralization [29]. The authors concluded that these features were consistent with an overall deficit in osteoblast function.

In the current study, we have used a range of anatomical, structural and biomechanical testing methods to investigate the biomechanical and material properties of the long bones in mice harbouring a functional knockout of *Mecp2*. Additionally, we have tested the reversibility of biomechanical phenotypes following un-silencing of the *Mecp2* gene.

## Material and methods

### Experimental animals

**Fig. 1:**
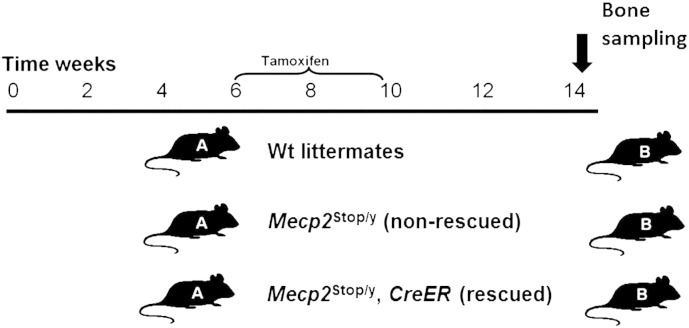
Experimental design of tamoxifen regime (rescuing) of *Mecp2*^stop/y^, *CreER*. Experimental design of the current study showing treatment (A) and sampling phases (B) in male mouse comparison cohorts. Wild-type (Wt) , *Mecp2*^stop/y^ (non rescue) and *Mecp2*^stop/y^, *CreER* (rescue) were given one injection of tamoxifen (100 mg/kg) per week for 3 weeks (age 6–8 weeks) then followed by 4 daily injections in consecutive days in the 4th week (age 9 weeks). Mice were then culled at 14 weeks and bones sampled for imaging, histology and biomechanical testing.

*Mecp2*^stop/+^ mice in which the endogenous *Mecp2* allele is silenced by a targeted stop cassette (*Mecp2*^tm2Bird^, Jackson Laboratories Stock No. 006849) were crossed with hemizygous *CreER* transgenic mice (*CAG-Cre*/*ESR1*, Jackson Laboratories Stock No. 004453) to create experimental cohorts [30]. A breeding strategy of crossing C57BL6/J/CBA F1 animals and using the F2 offspring was adopted as described previously [30]. The genotype of the mice was determined by polymerase chain reaction (PCR) [26]. Mice were housed in groups with littermates, maintained on a 12-h light/dark cycle and provided with food and water ad libitum. Experiments were carried out in accordance with the European Communities Council Directive (86/609/EEC) and a project licence with local ethical approval under the UK Animals (Scientific Procedures) Act (1986). The unsilencing of the *Mecp2* (removal of stop cassette, henceforth known as rescue mice) was achieved by tamoxifen (100 mg/kg) administered via intraperitoneal injection following regime described previously [30]. Briefly, male mice (wild-type, *Mecp2*^stop/y^ and *Mecp2*^stop/y^, *CreER* (Rescue)) were given one injection of tamoxifen (100 mg/kg) per week for 3 weeks (age 6–8 weeks) followed by 4 daily injections in consecutive days in the 4th week (age 9 weeks). Mice were then culled at 14 weeks (Fig. 1). Female mice display a more delayed onset RTT-like phenotype and were given an equivalent tamoxifen treatment regimen at 18 months of age (3 weekly followed a 4 daily injections) before being culled at 20 months. Wild type control mice were treated with tamoxifen in parallel with their littermates. Samples from the same age-matched cohorts were used for imaging, biomechanical and histological tests. Mice were culled by cervical dislocation and stored frozen at − 20 °C for biomechanical studies. For histological studies, mice were deeply anaesthetized with pentobarbitone (50 mg/kg, intraperitoneally) and transcardially perfused with 4% paraformaldehyde (in 0.1 M phosphate buffer, pH 7.4). To establish MeCP2 expression in bone tissues, we used a MeCP2-GFP reporter line as described previously [31] and with sections imaged by laser scanning confocal microscopy (Bio-Rad Radiance 2100, UK).

### Specimen preparation and morphometric measurements

Both right and left femurs and tibias along with the 5th lumbar vertebrae from each mouse were carefully dissected out. Femur and tibia whole bone wet weight measurements were taken using an analytical balance (APX60, Denver Instruments, UK). The femur and tibia were imaged using a WolfVision Visualizer VZ9.4F (WolfVision Ltd., Maidenhead, UK) and gross lengths were measured using Axiovision 4.8 Software (Carl Zeiss Ltd., Cambridge, UK). Femoral length measurements were taken from the proximal aspect of the greater trochanter to the distal end of bones, along the line of the shaft. Tibial length measurement was taken from the proximal aspect of the head of the tibia to the distal most aspect of the medial malleolus. Samples were then stored at − 20 °C in 0.1 M phosphate buffer prior to further testing. Right femurs were used for mechanical testing (the proximal part for the femoral neck test, the midshaft for microindentation) and left femurs were used for the bone histology (the proximal femur for sirius red and TRAP staining, the distal femur for scanning electron microscopy). Right tibias were used for μCT and three-point bending tests. The 5th lumbar vertebrae were used for bone mineral density and trabecular bone structure measures. The right humeri were used for analysis of the bone mineral structure using Small Angle X-ray Scattering (SAXS).

### Micro-computed tomography (μCT)

Tibias and lumbar 5 vertebras were scanned with a SKYSCAN® 1172/A μCT Scanner (Bruker, Belgium). Images were reconstructed and analysed using the NRecon 1.6.6.0 and CT-Analyser 1.8.1.3 software (Bruker, Belgium). For the tibia, 34 μm resolution was used and the X-ray tube was operated at 54 kV and 185 μA. Bone samples were scanned in physiological 0.9% NaCl solution. For cortical bone parameter analyses, tibial 2 mm midshaft regions of interest (ROI) were selected, starting from the anatomical point of the tibiofibular junction in each specimen. A lower grey threshold value of 113 and upper grey threshold value of 255 was used as thresholding values in each cortical bone sample. Individual two dimensional object analyses were performed on six sections per specimen within each comparison genotype group to calculate the inner and outer perimeters of bone. Three dimensional analyses were further used to calculate cortical thickness, marrow area, cortical area, total area, bone volume and second moment of area.

Lumbar 5 vertebrae were scanned at a resolution of 5 μm. The X-ray tube was operated at 41 kV and 240 μA. A lower grey threshold value of 81 and upper grey threshold value of 252 was used as thresholding values in each trabecular bone sample. A cylindrical region of interest (150 slices or 0.774 mm) was selected from the centre of each vertebral body. Calibration of the standard unit of X-ray CT density from Hounsfield units to volumetric bone mineral density (vBMD) was conducted. ROIs were analysed for the following parameters: trabecular thickness, trabecular separation, trabecular bone volume, trabecular porosity, as well as degree of anisotropy (DA) and structure model index (SMI).

### Mechanical tests

Right tibial and femoral shafts from each comparison group were subjected to mechanical testing (three point bending and microindentation tests respectively) after the μCT. The mechanical tests were designed to test the cortical part of bone. The tests were performed using a Zwick/Roell z2.0 testing machine (Leominster, UK) with a 100 N load cell [32].

### Three-point bending test

Tibias were placed on the lower supports, at 8 mm separation, with the posterior surface of the tibia facing down. Load was applied with a loading rate of 0.1 mm s^− 1^ on the shaft of the tibia using the Zwick/Roell testing machine until the fracture occurred (Fig. 3A). Data were analysed to determine values of stiffness, ultimate load and Young's modulus using the following formula:

(1) Young'smodulus=stiffτ⋅Ls348⋅I

where stiffτ is the stiffness. Ls is the separation of the supports and I is the second moment of area of the tibias. The stiffness was calculated by measuring the slope of the force-displacement graph and the ultimate load by measuring the maximum force that the bone was able to resist. The second moment of area was calculated using the microCT data and ImageJ software v1.47 and the plug-in Bone J.

### Micro indentation hardness test

The micro indentation hardness test was performed on equivalent transverse distal mid-shaft sections of right femur for each mouse/genotype. Bone sections were air dried and embedded in metallurgical mounting resin (EPO Set Resin, Meta Prep, UK) and the moulds allowed to solidify at room temperature for 24 h. The bone cross-section surface was subsequently polished using silicon carbide papers with decreasing grain size (240, 400, 600, 800, 1200) and diamond paste (15, 6 and 1 μm) to produce a smooth surface.

After the sample preparation, micro hardness testing was performed using a Wilson Wolport Micro-Vickers 401MVA machine (UK), with an applied load of 25 g for 100 sec. The bone was tested at seven points for each specimen (Fig. 4A). The Vickers pyramid hardness number (HV) was calculated using Eq. 2 where the load (L) is in grammes force and the average length of the two diagonals (D) is in millimetres:

(2) $$HV\;=\;1.854\frac{L}{D2}\text{.}$$

### Femoral neck fracture test

The femoral neck fracture test was used to test the mechanical properties of the femoral neck. The shaft of the femur was fixed in a mechanical chuck and placed in the Zwick/Roell mechanical testing machine. The bone was clamped at a 9° angle lateral to the vertical axis of the bone as described previously [32]. Load was applied to the femoral head until fracture occurred. Stiffness was obtained from the slope of the force-displacement curve and the ultimate load obtained was from the maximum force that the bone was able to resist.

### Bone histology

Proximal parts of the femurs were decalcified in 11% EDTA (pH 8.0, 5 N NaOH) for 14 days. Samples were embedded in paraffin wax and 5 μm longitudinal sections were cut on a microtome (Leica RM2035, Milton Keynes, UK). Alternate sections were stained with sirius red staining for collagen content and Tartrate-resistant acid phosphatase (TRAP) staining for osteoclasts. The sirius red staining was completed using the picro-sirius red method as described [33] followed by counterstaining with haematoxylin. To standardize staining, all sections were stained in a single batch. To assess the collagen content, sections from the proximal femur shaft were stained with sirius red and bright field images collected (n = 5 for each mouse) using an Axioskop50 microscope with a 40× objective (Zeiss, Cambridge, UK) and Carl Zeiss AxioCam MRc camera (Zeiss, Cambridge, UK). Five regions of interest (approximately 219 μm × 164 μm), were selected for quantification, and averages per section were taken as the final measures for each genotype. The % area of red pixels corresponding to collagen fibres, relative to total tissue area, was estimated using a colour segmentation plugin in ImageJ (Biomedical Imaging Group, EPFL, Switzerland: http://bigwww.epfl.ch/sage/soft/colorsegmentation/) using independent colour channels and the K-means algorithm.

### Scanning electron microscopy

Distal femurs were sectioned transversely just above the condyles and stored in 2.5% paraformaldehyde in 0.1 M sodium phosphate buffer (pH 7.4) at 4 °C for 48 h. Adherent soft tissue was removed by immersion in 3% hydrogen peroxide solution for 48 h. After rinsing with distilled water, specimens were defatted in 50:50 methanol/chloroform for 24 h at room temperature and transferred to a 5% trypsin solution (0.1 M PB, pH 7.4) at room temperature for 48 h. After cleaning with distilled water, specimens were desiccated prior to preparing on a sputter coater (Polaron E5000, East Sussex, UK). Images were obtained using a scanning electron microscope (Stereoscan 250 MK3, Cambridge, UK).

### Small Angle X-ray Scattering

Small Angle X-ray Scattering (SAXS) was used to assess the nano-scale bone mineral structure of the cortical bone in the humerus of the female mice. Five right humeri from each group of the female mice were formalin fixed, dehydrated in a series of increasing concentration alcohol solutions and embedded in methylmethacrylate resin. A transverse slice was cut from the mid shaft and polished down to 100 μm thickness.

The I911-SAXS beamline of the MAX II ring (1.5 GeV) at the MAX IV Laboratory (Lund University, Lund) was used [34]. A monochromatic beam of wavelength 0.91 Å was obtained using a Si(111) crystal and collimated down to 100 μm by 100 μm at the sample. The *q*-range measured was 0.01–0.30 Å^− 1^. Measurements were conducted with the samples mounted on an x–y motorised stage and a step size of 100 μm with an exposure time of 5 s at each point was used to scan the cross-section of the bone [35]. The detector used was a PILATUS 1 M (Dectris Ltd.). The mineral plate thickness, predominant orientation and degree of orientation of the mineral crystals were calculated for each scattering image as described earlier [35–37]. Only scattering images where the signal level indicated the presence of cortical bone were analysed.

### Data analysis and statistics

Unless states otherwise, all data is given as mean ± standard deviation (S.D.). For statistical analysis of imaging, biomechanical and histological data, one way ANOVA with Tukey's post hoc test were conducted using Prism 5.0 (Graphpad, USA) with alpha being 0.05.

## Results

**Fig. 2:**
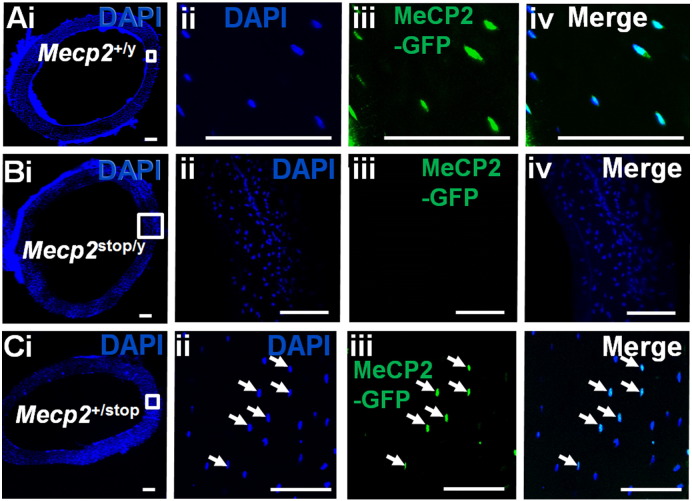
MeCP2 is expressed widely in bone tissues. (A) Low power and (ii–iv) high power micrographs of transverse sections taken from mid shaft mouse femur showing GFP expression in all DAPI-labelled nuclei in a male *Mecp2*^+/y^ mouse in which the native MeCP2 is tagged with a C-terminal GFP. Note that MeCP2 is restricted to the nucleus of osteocytes as indicated by the complete overlap with DAPI staining but present in all nuclei. (B) GFP expression is not observed in mice in which MeCP2 expression is functionally silenced by a neo-stop cassette. (C) Low power (i) and (ii–iv) high power micrographs showing mosaic expression of GFP-tagged MeCP2 protein in ~ 50% of DAPI positive nuclei in a female heterozygous *Mecp2*^+/stop^ mouse in which one *Mecp2* allele is functionally silenced. All scale bars: 100 μm.

MeCP2 protein is particularly abundant in post-mitotic cells of the brain, but is also widely expressed throughout the body [7,9,38]. In order to confirm that bone cells express MeCP2 we used a reporter mouse line in which MeCP2 expresses a C-terminal GFP tag [31]. We observed that all bone cells express nuclear GFP fluorescence in both wild type male (Fig. 2A) and female mice (data not shown). In contrast, GFP fluorescence is absent in hemizygous *Mecp2*^stop/y^ mice (Fig. 2B), in which *Mecp2* is silenced by a stop cassette, and is observed in ~ 50% of nuclei in heterozygous *Mecp2*^+/stop^ mice in which one *Mecp2* allele is silenced to mimic the mosaic expression pattern seen in human female Rett syndrome [26,30] (Fig. 2C). In order to determine any gross skeletal abnormalities caused by MeCP2 deficiency, the tibia and femur of male *Mecp2*^stop/y^ mice together with wild-type littermates were examined for gross morphometric and weight measures (Table 1). No difference in whole body weights was observed between genotypes in male mice (Wt = 31.88 ± 3.85 g; *Mecp2*^stop/y^ = 28.14 ± 4.07 g; *Mecp2*^stop/y^, *CreER* = 27.74 ± 2.68 g; n = 5 per genotype; *p* < 0.05, ANOVA with Tukey's post hoc test) or in the female comparison genotypes (Wt = 32.72 ± 5.59 g; *Mecp2*^+/stop^ = 41.70 ± 7.15 g; *Mecp2*^+/stop^, *CreER* = 39.47 ± 9.77 g; n = 5 per genotype; *p* < 0.05, ANOVA with Tukey's post hoc test). *Mecp2*^stop/y^ mouse femurs showed a significantly reduced weight in comparison with wild-type (Wt) littermate controls and *Mecp2*^stop/y^, *CreER* (Wt = 51.90 ± 3.77 mg; *Mecp2*^stop/y^ = 44.80 ± 3.41 mg; *Mecp2*^stop/y^, *CreER* = 51.80 ± 5.87 mg; n = 5 per genotype; *p* < 0.05, ANOVA with Tukey's post hoc test). A similar trend was observed in *Mecp2*^stop/y^ mouse tibias, weight measures (Wt = 55.50 ± 2.11 mg; *Mecp2*^stop/y^ = 49.20 ± 1.21 mg; *Mecp2*^stop/y^, *CreER* = 52.12 ± 2.96 mg; n = 5 per genotype; *p* < 0.05, ANOVA with Tukey's post hoc test). There was an accompanying reduction in tibial length (*p* < 0.01), but no significant difference in femoral length between groups (*p* > 0.05) (Table 1). Female *Mecp2*^+/stop^ showed no significant difference in tibial weight or length in comparison with Wt or *Mecp2*^+/stop^, *CreER* mice in which *Mecp2* was reactivated 7 weeks prior to testing (all *p* > 0.05) (Table 1).

### Biomechanical testing revealed genotype differences in bone properties

In order to explore possible differences in the mechanical and material properties of MeCP2-deficient bone, tests were applied to femurs and tibias isolated from male hemizygous *Mecp2*^stop/y^ mice and from female heterozygous *Mecp2*^+/stop^ mice together with their wild-type and treated (unsilenced *Mecp2*) littermates.

### Three point bending test

**Fig. 3:**
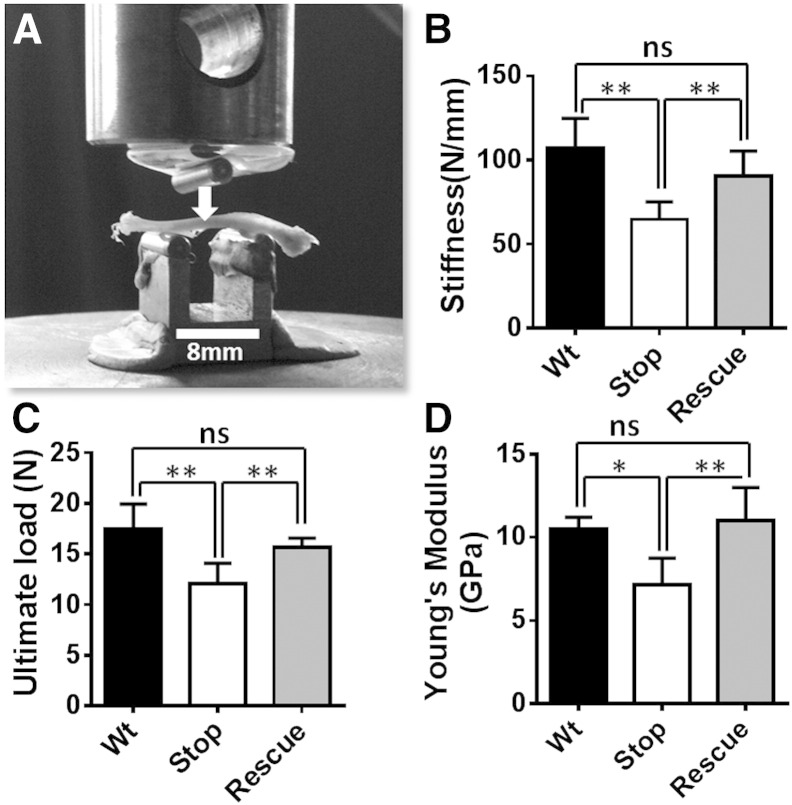
Three-point bending test reveals mechanical deficits in the tibia of male MeCP2-deficient mice. (A) Mouse tibia were placed on the posterior surface across two supporting bars with a distance of 8 mm apart and a load was applied to the anterior surface of shaft until the bone fractured. In male mice the measures of (B) cortical bone stiffness (*p* < 0.01; one way ANOVA with Tukey's post hoc test; n = 5 tibia per genotype), (C) ultimate load (*p* < 0.01) and (D) Young's modulus (*p* < 0.05) were significantly reduced in *Mecp2*^stop/y^ (Stop) mice as compared to wild-type (Wt), and genetically rescued *Mecp2*^stop/y^; *CreER* (Rescue) mice. Abbreviation: ns = not significant; *p < 0.05, **p < 0.01. Plots show mean ± S.D.

In order to test the mechanical properties (stiffness, ultimate load and Young's modulus) of compact bone a three point bending test was applied to tibial shafts (Fig. 3A). It revealed a reduced structural stiffness (Fig. 3B; Wt = 106.8 ± 17.88 N/mm; *Mecp2*^stop/y^ = 64.7 ± 10.50 N/mm; *Mecp2*^stop/y^, *CreER* = 90.7 ± 14.83 N/mm, n = 5 per genotype; *p* < 0.01, ANOVA with Tukey's post hoc test), ultimate load (Fig. 3C; Wt = 17.50 ± 2.45 N; *Mecp2*^stop/y^ = 12.09 ± 1.94 N; *Mecp2*^stop/y^, *CreER* = 15.7 ± 0.08 N; n = 5 per genotype; *p* < 0.01, ANOVA with Tukey's post hoc test) and Young's modulus (Fig. 3D; Wt = 10.52 ± 0.69 GPa; *Mecp2*^stop/y^ = 7.14 ± 1.61 GPa; *Mecp2*^stop/y^, *CreER* = 11.92.4 ± 2.06 GPa; n = 5 per genotype; *p* < 0.01, one way ANOVA with Tukey's post hoc test) measures in male *Mecp2*^stop/y^ mice. Samples from *Mecp2*^stop/y^, *CreER* mice revealed that stiffness, ultimate load and Young's modulus measures were not different from wild-type values (Fig. 3B–D).

**Fig. 4:**
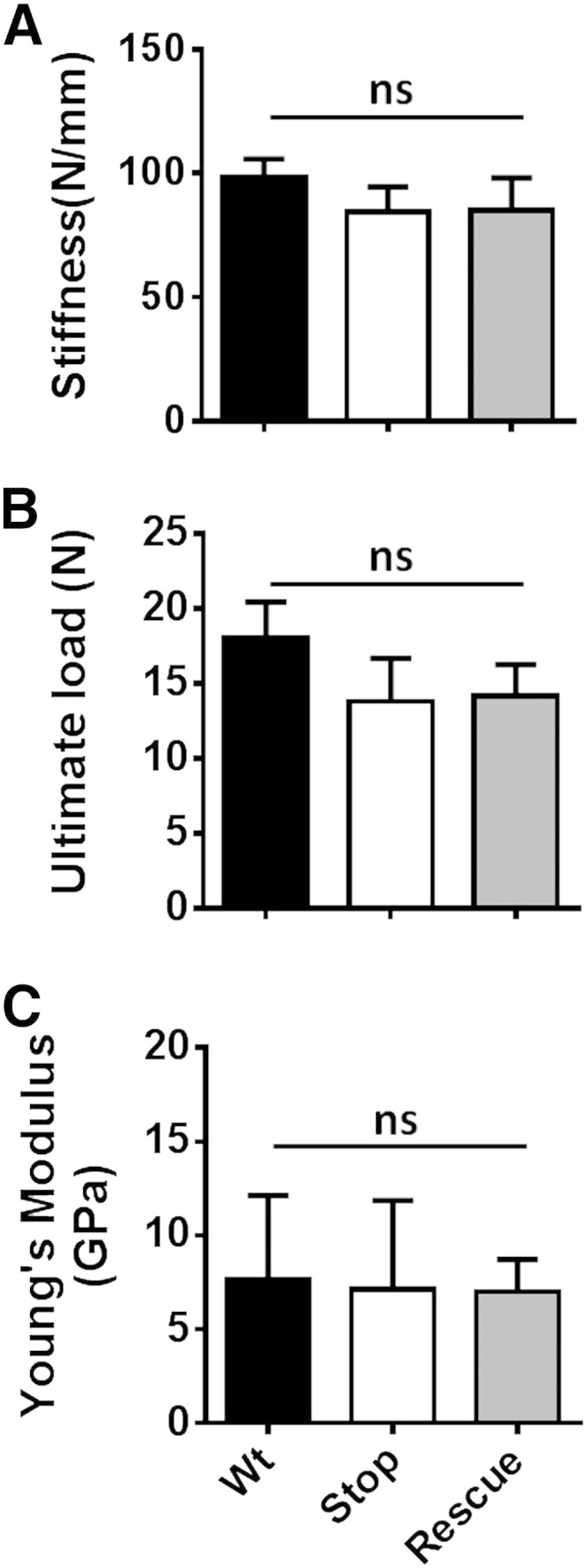
Three-point bending test reveals no mechanical deficits in the tibia of heterozygous *Mecp2*^+/stop^ mice. Bar plot showing measures of (A) cortical bone stiffness, (B) ultimate load and (C) Young's modulus in female wild-type (Wt), *Mecp2*^stop/+^ (Stop) and *Mecp2*^stop/+^ (Rescue) mice (one way ANOVA with Tukey's post hoc test; n = 3–5 per genotype). Abbreviation: ns = not significant; plots show mean ± S.D.

The same tests when conducted on tibias from female *Mecp2*^+/stop^ mice showed no significant difference in stiffness, load or Young's modulus (Fig. 4; all *p* > 0.05).

### Microindentation hardness test

**Fig. 5:**
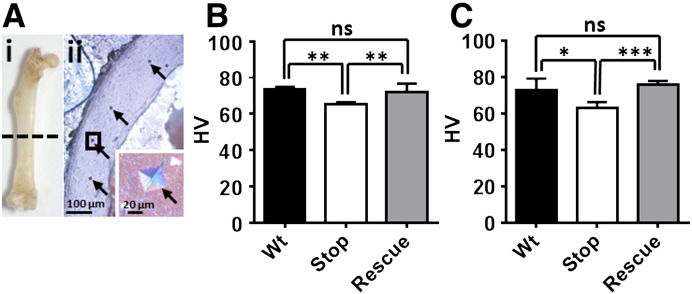
Microindentation hardness test reveals a significant but reversible reduction in cortical bone hardness in MeCP2-deficient mice. (A). Mouse femur (i) was sectioned at mid-shaft, mounted, polished and assessed by microindentation (ii; indentation marks shown by arrows). B. Microindentation test in male mice revealed a significant reduced cortical bone hardness in *Mecp2*^stop/y^ (Stop) mice when compared with the wild-type (Wt) controls (*p* < 0.05, one way ANOVA with Tukey's post hoc test, n = 5 femurs per genotype). In contrast, microhardness measures in rescued *Mecp2*^stop/y^, *CreER* (Rescue) mice were not different from controls (*p* > 0.05; n = 5 femurs). (C) Microindentation hardness test results in female mice showed a similar pattern with reduced cortical bone hardness in *Mecp2*^+/stop^ (Stop) mice when compared with wild-type (Wt) controls and rescued mice (n = 3–5 femurs per genotype, one way ANOVA with Tukey's post hoc test). Abbreviations: ns = not significant (* = *p* < 0.05, ** = *p* < 0.01, *** = *p* < 0.001). Plots show mean ± S.D.

To assess the material hardness of bone, mid-shaft femur was dissected from each mouse and subjected to micro indentation testing (Fig. 5A). Results from male mice showed significantly reduced bone hardness in *Mecp2*^stop/y^ mice compared to wild-type littermates (Fig. 5B). Moreover, tamoxifen-treated *Mecp2*^stop/y^, *CreER* mice did not differ significantly from wild-type and showed a significant treatment effect when compared with the *Mecp2*^stop/y^ cohort (Fig. 5B; Wt = 73.7 ± 1.3 HV, *Mecp2*^stop/y^ = 65.4 ± 1.2 HV, *Mecp2*^stop/y^, *CreER* = 72.1 ± 4.7 HV, n = 5 per genotype, *p* < 0.01, ANOVA with Tukey's post hoc test). A significant deficit in bone hardness was also observed in female *Mecp2*^+/stop^ femurs (Fig. 5C; Wt = 72.8 ± 6.3 HV, *Mecp2*^+/stop^ = 63.2 ± 3.0 HV, *Mecp2*^+/stop^_,_ *CreER* = 75.7 ± 2.2 HV, n = 3–5 per genotype; p < 0.01, ANOVA with Tukey's post hoc test). Again, rescue mice showed a significant treatment effect and measures were not found significantly different from wild-type.

### Femoral neck fracture test

**Fig. 6:**
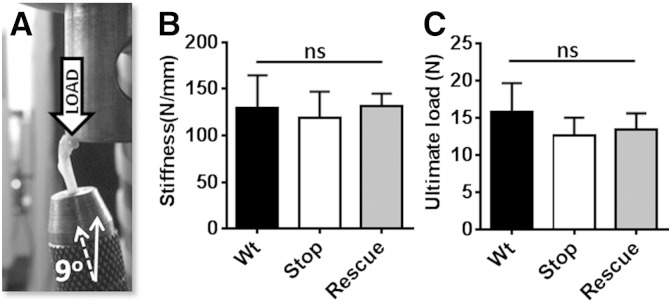
Femoral neck test revealed no alteration in bio-mechanical properties in MeCP2-deficient mice. (A) Image showing femoral neck test in which load was applied downward onto the femoral head until the femoral neck fractured. Results in male mice showed no significant difference in measurements of (B) bone stiffness or (C) ultimate load between wild-type (Wt), *Mecp2*^stop/y^ (Stop) and *Mecp2*^stop/y^, *CreER* (Rescue) mice (*p* > 0.05; one way ANOVA; n = 5 per genotype). Plots show mean ± S.D. Abbreviations: ns = not significant.

**Fig. 7:**
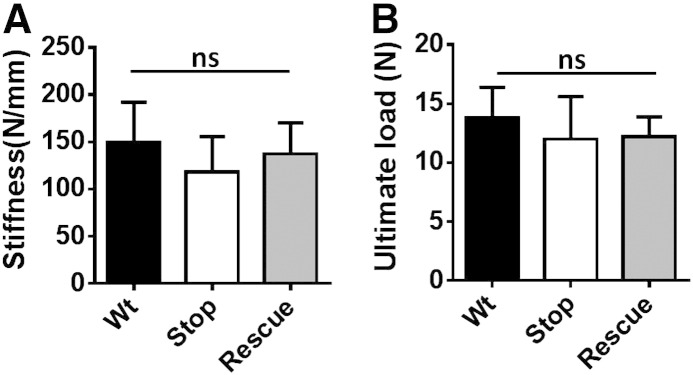
Femoral neck test revealed no alteration in bio-mechanical properties in female MeCP2-deficient mice. Bar plots showing no significant difference in measures of (A) bone stiffness or (B) ultimate load between wild-type (Wt), *Mecp2*^stop/+^ (Stop) and *Mecp2*^stop/+^, *CreER* (Rescue) mice (all *p* > 0.05; ANOVA; n = 3–5 per genotype). Plots show mean ± S.D. Abbreviations: ns = not significant.

This test was conducted to assess possible group differences in the mechanical properties of the femoral neck (Fig. 6A). In male mice, no significant differences were observed in stiffness (Fig. 6B; stiffness: Wt = 130 ± 35.1 N/mm; *Mecp2*^stop/y^ = 119 ± 28.2 N/mm; *Mecp2*^stop/y^, *CreER* = 131 ± 13.9 N/mm, n = 5 per genotype; *p* > 0.05, ANOVA with Tukey's post hoc test), or ultimate load (Fig. 6C; Wt = 15.9 ± 3.9 N; *Mecp2*^stop/y^ = 12.6 ± 2.4 N; *Mecp2*^stop/y^, *CreER* = 13.4 ± 2.2 N, n = 5 per genotype, *p* > 0.05, ANOVA with Tukey's post hoc test). Similar findings were obtained in the female groups (Fig. 7).

### *Mecp2*^stop/y^ mice showed reduced collagen content and altered trabecular bone structure

**Fig. 8:**
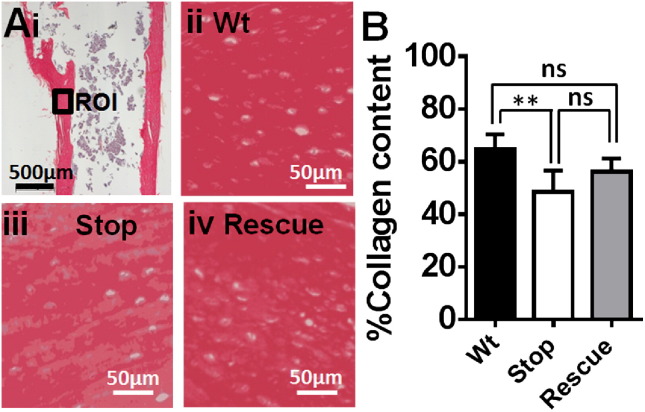
Sirius red staining revealed a reduced collagen content in MeCP2-deficient mice. (Ai) Longitudinal section of proximal femur stained with sirius red to reveal collagen. Regions of interest (ROI) from shaft of proximal femur were selected for quantification. Collagen content was measured in (ii) wild-type (Wt), (iii) *Mecp2*^stop/y^ (Stop) and (iv) *Mecp2*^stop/y^, *CreER* (rescue) comparison groups. (B) Bar chart showed that % Collagen content was reduced in *Mecp2*^stop/y^ (Stop) mice as compared to wild-type (Wt; *p* < 0.01). Abbreviations: ns = not significant (* = *p* < 0.05, ** = *p* < 0.01; one way ANOVA with Tukey's post hoc test). Plots show mean ± S.D.

Picrosirius red staining of the femur was used to assess collagen content (Fig. 8A) as described previously [39]. *Mecp2*^stop/y^ mice showed a significant decrease (− 24%) in collagen content compared to Wt mice (Fig. 8B; Wt = 65.1 ± 8.6%; *Mecp2*^stop/y^ = 48.8 ± 9.1%; *Mecp2*^stop/y^, *CreER* = 55.63 ± 11.4%; n = 10 per genotype, *p* < 0.01, one way ANOVA with Tukey's post hoc test).

TRAP staining was conducted to assess resorption activity (osteoclast number per bone surface), but showed no difference between genotypes (Wt = 12.61 ± 8.51; *Mecp2*^stop/y^ = 17.48 ± 6.13; *Mecp2*^stop/y^, *CreER* = 18.90 ± 4.61; n = 5 per genotype, *p* > 0.05, one way ANOVA with Tukey's post hoc test).

**Fig. 9:**
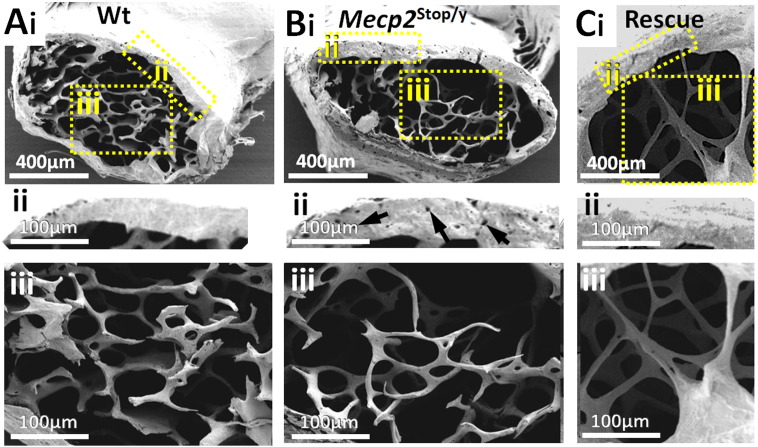
Scanning electron microscopy reveals pitted cortical bone and altered trabecular structure in distal femur of male MeCP2-deficient mice. Scanning electron micrographs of distal femur in (Ai) wild-type (Wt) and (Bi) *Mecp2*^stop/y^ (Stop). Higher powered images of cortical (ii) and metaphyseal (iii) regions (areas indicated in A) reveal a more porous structure in cortical bone (arrows in Bi indicate pores) and a sparse trabecular structure in *Mecp2*^stop/y^ mice when compared with representative with Wt controls. (Ci–iii) Representative micrograph from a *Mecp2*^stop/y^, *CreER* (Rescue) mouse.

Qualitative analysis using scanning electron microscopy (SEM) of the distal femur (n = 5 per genotype) revealed porous structure in cortical bone (3 of 5 mice) as well as alterations in the architecture of trabecular bone in *Mecp2*^stop/y^ mice (Fig. 9A–B). The central metaphyseal region in *Mecp2*^stop/y^ mice showed a sparse trabecular mass consisting of short, thin trabecular rod and plate structures. In contrast, a more robust trabecular structure, with a network of shorter and thicker rods and plates was found in wild-type control tissue (Fig. 9Ai–ii). The porosity and altered trabecular structure was less evident in rescued *Mecp2*^stop/y^, *CreER* mice (Fig. 9C). These features were investigated further and a quantitative manner using μCT (below). In contrast to the male mice, we did not observe overt tissues differences in heterozygous *Mecp2*^stop/+^ mice.

### Micro computed tomography (μCT) show altered trabecular and cortical bone parameters in *Mecp2*^stop/y^ mice

**Fig. 10:**
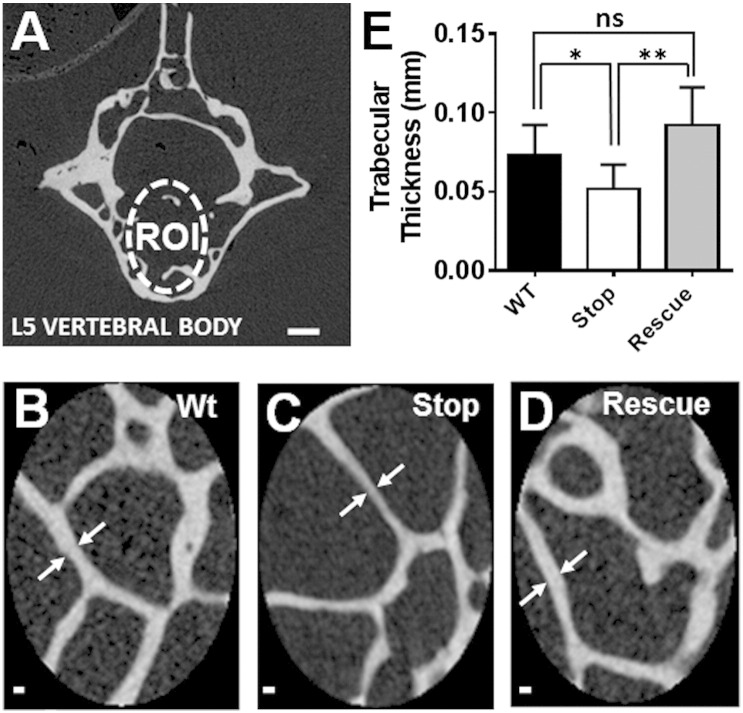
μCT scans of L5 vertebrae revealed thinner trabecular mass in MeCP2-deficient mice. (A) μCT image showing trabecular region of interest (ROI) selected within the 5th Lumbar vertebral body. (B–D) Micrographs showing representative trabecular samples from wild-type (Wt), *Mecp2*^stop/y^ (Stop) and *Mecp2*^stop/y^, *CreER* (Rescue) mice. (E) Bar plot showing quantitative analysis of trabecular thickness (arrows in B–D). Note the reduced thickness in *Mecp2*^stop/y^ samples (*p* < 0.05; n = 7 per genotype). Scale bar: A, 200 μm; B–D, 50 μm. Abbreviations: ns = not significant (* = *p* < 0.05, ** = *p* < 0.01; one way ANOVA with Tukey's post hoc test). Plots show mean ± S.D.

Three dimensional μCT analysis was performed to obtain a quantitative measure of trabecular architecture in wild-type, *Mecp2*^stop/y^ and *Mecp2*^stop/y^, *CreER* mouse lumbar 5 (L5) vertebrae (Fig. 10A). A significant reduction of L5 trabecular thickness (~ 30%) was observed in *Mecp2*^stop/y^ mouse tissues compared to the wild-type control. Interestingly *Mecp2*^stop/y^, *CreER* mouse L5 μCT results, showed a significant increase (+ 80%, *p* < 0.01) in trabecular rod and plates thickness compared to *Mecp2*^stop/y^ mice (Fig. 10B–E; Wt = 0.073 ± 0.01 mm; *Mecp2*^stop/y^ = 0.051 ± 0.02 mm; *Mecp2*^stop/y^, *CreER* = 0.09 ± 0.02 mm; n = 7 per genotype; *p* < 0.01, ANOVA with Tukey's post hoc test). No significant differences were observed in trabecular separation, trabecular bone volume, trabecular porosity, bone mineral density (BMD), degree of anisotropy (DA) and structure model index (SMI) between genotypes (Table 2). μCT analysis of tibia showed a significant difference in cortical bone thickness, outer perimeter length, inner perimeter length, marrow area, total area and bone volume in *Mecp2*^stop/y^ mouse compared to wild-type controls (*p* < 0.05, n = 7 per genotype, ANOVA with Tukey's post hoc test). Bone perimeter, total area and bone volume values remained decreased in *Mecp2*^stop/y^, *CreER* mice. These data are summarized in Table 2. μCT analysis of female tibia showed no difference in cortical bone parameters between the three comparison genotypes (data not shown).

### SAXS shows negligible changes in mineral particle size and orientation

No differences could be seen between the mineral plate thickness in the three groups when calculated using the Bünger method (Wt = 3.24 ± 0.26 nm, Stop = 3.20 ± 0.15 nm and Rescue = 3.31 ± 0.12 nm). Similarly the degree of orientation (where 0 = no alignment and 1 = perfect alignment) showed no significant difference (Wt = 0.572 ± 0.009, Stop = 0.575 ± 0.005 and Rescue = 0.576 ± 0.014). In general the mineral orientation was circumferential, thus making an average mineral orientation calculation irrelevant. However, when the data was considered in detail, the Stop bones showed more pixels where the scattering intensity signal was below the level normally considered to be cortical bone, indicating the decreased organisation of the tissue.

## Discussion

The results of our current study show that MeCP2-deficiency in mice results in altered material, structural and functional properties of bone tissues. There is a growing awareness of skeletal anomalies (low energy fractures, scoliosis, kyphosis) in Rett patients [8,10,11,13–15,18–21,40–56] and the aim of the current project was to assess further the nature and tractability of bone phenotypes. Our morphometric analysis showed a reduction in long bone weight and, in the case of the tibia, a reduction in length in *Mecp2*^stop/y^ mice. Such findings are generally consistent with those reported in the *Mecp2*-null mouse by O'Connor and colleagues [29] and reflect the fact that MeCP2-deficient male mice are generally smaller and display a kyphotic appearance [25]. However, the *Mecp2-stop* line used in the current experiments did not show differences in bodyweight. Furthermore, our study showed that adult female heterozygous *Mecp2*^+/stop^ mice did not show differences in gross tibia and femur length/weight measures. Female *Mecp2*^+/stop^ mice are a gender appropriate and accurate genetic model of RTT yet show more subtle and delayed onset (4–12 months) neurological features [26] compared to hemizygous male mice.

A major finding of the current study was the demonstrated robust deficits in mechanical properties and micro-hardness of bone seen in the male *Mecp2*^stop/y^ mice. Such deficiencies in mechanical and material properties were profound (32.1% reduction in stiffness in the three point bending test; 31% reduction in maximum load and 12.3% reduction in microhardness). Males with Rett syndrome are extremely rarely diagnosed, possibly due to the early death of these patients, prior to neurological diagnosis of Rett syndrome and there are no clinical reports of bone phenotypes in males. However the above findings could nevertheless explain the occurrence of low energy fractures reported in female Rett syndrome patients [15–17,50,57]. Whilst *Mecp2*-knockout mice display many of the neurological features seen in Rett patients (motor impairments and abnormal breathing), there are important differences in Rett-like phenotypes in mice and those observed in patients. In particular, females with RTT develop symptoms as young children whereas heterozygous *Mecp2*-KO mice develop overt phenotypes late on in adulthood and they are generally much milder. For instance, spontaneous seizures and autonomic abnormalities are common in patients but rarely seen in mice. As such RTT-like phenotypes in mice are considered much less severe and in this respect is could be argued that the RTT-like phenotypes seen in male *Mecp2*-KO mice are somewhat closer to the clinical picture (juvenile onset of symptoms which then become very severe) although, like RTT in male patients, the consequence of mutation/KO is invariably fatal beyond early/mid adulthood. Whilst we have not observed overt signs of spontaneous fractures in experimental colonies of mice, such a magnitude of reduced bone stiffness and load properties could mirror the 4 times increased risk of fracture in Rett patients compared to the population rate [15]. That a similar reduction in microhardness (and a trend towards reduced biomechanical properties) was seen in female mice (Figs. 4, 5 and 7) that are heterozygous and mosaic for the mutant allele, demonstrate that the bone deficits are not restricted to the more severe male RTT-like phenotype but are seen in a gender and MeCP2 expression pattern appropriate model of RTT, albeit one that is milder than RTT in human females. Analysis of femoral neck fracture showed no difference between genotypes. It is possible that the complex microstructure of bone in the femoral neck (cf. the simple cortical shaft geometry) is a confounding factor and limits the sensitivity of this test. Indeed, we also noted greater variance in this test than in the other biomechanical tests which may also limit our ability to resolve subtle changes in this parameter. However, it is also possible that any deficits are too subtle to be detected given the power of the current study. Whilst group sizes of the order used in the current experiments enable the unambiguous detection of overt neurological phenotypes, it is likely that bone phenotypes are more subtle and that much larger group sizes would be required to detect subtle changes in histological and biomechanical phenotypes, especially in heterozygous *Mecp2*^+/−^ mice.

An important finding of the current study and one with therapeutic implications is that the observed deficits in cortical bone material and biomechanical properties were rescued by delayed postnatal activation of the *Mecp2* gene. This finding mirrors the improvements seen in multiple non-bone phenotypes seen in the *Mecp2*^stop/y^ mice after delayed activation of the *Mecp2* gene including survival, normalized bodyweight, locomotor and behavioural activities [26,30]. These results suggest that the bone abnormalities present in RTT patients may be at least partially reversible using gene-based therapies that are currently being developed [58,59]. However, it is also possible that significant amelioration of bone phenotypes may also be achieved using pharmacological strategies. Of particular importance for this approach is to identify the mechanisms by which MeCP2 deficiency results in altered bone properties. Whilst we show that MeCP2 is expressed in osteocytes, the protein is widely expressed throughout the body and it is possible that metabolic and endocrine perturbations elsewhere in the body also impact on bone homeostasis.

The precise molecular role of MeCP2 in the nucleus remains unclear [4,6,60,61], but it is generally considered to regulate gene expression. As collagen is the most abundant gene product and structural determinant in bone, we conducted an initial analysis of collagen content and distribution using sirius red staining. The decreased levels of intense sirius red stain observed in the MeCP2-deficient mice is consistent with reduced collagen [56] and the patches of reduced staining resemble those features characteristic of early osteoporosis [17]. Indeed, the osteopathic features of RTT (minimal bone deformity, low energy bone fractures, and tendency towards spinal curvature) are similar to those reported in collagen type 1 genetic disorder (osteogenesis imperfecta; brittle bone disease) [62] pointing towards the possible importance of collagen defects in RTT. In addition to structural protein, we also investigated the resorptive properties of the bone in terms of TRAP staining. The lack of any difference in osteoclast number between genotypes is consistent with a previous report [29] and suggests the possible absence of any primary defect in bone remodelling. Similarly, the limited effects seen in SAXS analysis the bone at the nanometre scale indicates minimal change in the mineral phase of bone, but there is an indication that the amount and slightly more macroscale tissue organisation is affected.

Despite this finding, qualitative analysis by scanning electron microscopy did reveal altered trabecular architecture (widely spaced and thin trabeculae) in *Mecp2*^stop/y^ mice, consistent with the overall osteoporotic picture and suggesting clear structural differences between genotypes which would be consistent with reduce bone integrity. The cortical area surrounding the central rod and plate mass showed characteristic pits in *Mecp2*^stop/y^ which were much less numerous in wild-type controls. These could result from increased nutrient foramina or poorly laden osteoporotic bone due to osteoblast dysfunction. The quantitative μCT findings from only the trabecular portion of L5 vertebrae were carried out and the results are consistent with the SEM findings in that the trabecular thickness was significantly reduced in *Mecp2*^stop/y^ mice. As with the functional tests on long bones, trabecular thickness was normalized to wild type levels upon unsilencing *Mecp2* in the *Mecp2*^stop/y,^ *CreER* cohort, indicative of a pronounced phenotypic rescue and evidence of structural remodelling upon activation of MeCP2 analogous to structural remodelling demonstrated in the brain [30].

A surprising finding of the current study was the absence of any bone mineral density (μCT) differences between genotypes. Reduced bone mass is commonly associated with osteoporotic phenotypes [50,63–65] and bone mineral content differences have been reported in *Mecp2*-null mice [29]. The lack of observed differences (weight, length, density) in the current study may be due to differences between mouse models (strain, mutation type, age). Both the synthesis of collagen and its mineralization are crucial for the bone tissue biomechanical properties and % collagen content is an important marker of biomechanical strength of bone, independent of the bone density [66]. Given this, it is possible that the functional deficits identified in the current study are due to abnormalities in structural proteins of bone tissue rather than the gross mineral content. We aim to resolve this issue in future studies by exploring further the nanostructure of cortical bone as well as individual structural proteins.

## Conclusions

In this study we have identified a range of anatomical, biomaterial and biomechanical abnormalities in bone of MeCP2-deficient mice and have shown that many of these features are potentially reversible by reactivating the *Mecp2* gene, even in fully adult mice. These results suggest that bone phenotypes may be important yet tractable features of RTT and should be considered in future studies aimed at developing pharmacological and generic interventions for the disorder.

## Figures and Tables

**Table 1:** Morphmetric measurements of male and female mice.

	Male mouse			Female mouse		
	Wild-type	*Mecp2*^stop/y^	*Mecp2*^stop/y^, *CreER*	Wild-type	*Mecp2*^+/stop^	*Mecp2*^+/stop^, *CreER*
Femur weight (mg)	51.90 ± 3.77	44.80 ± 3.41⁎, a	51.80 ± 5.87⁎, b	84.66 ± 9.47	79.50 ± 8.64	87.40 ± 6.99
Femur length (mm)	13.78 ± 0.23	13.62 ± 0.22	13.70 ± 0.20	14.15 ± 0.57	13.45 ± 0.87	14.02 ± 0.02
Tibia weight (mg)	55.50 ± 2.11	49.20 ± 1.21⁎⁎, a	52.12 ± 2.96	68.84 ± 4.08	66.60 ± 2.98	68.80 ± 10.40
Tibia length (mm)	17.66 ± 0.84	15.94 ± 0.48⁎⁎, a	16.88 ± 0.51	16.33.0 ± 0.66	15.90 ± 1.52	15.31 ± 0.59
Average body weights (g)	31.88 ± 3.85	28.14 ± 4.07	27.74 ± 2.68	32.72 ± 5.59	41.70 ± 7.15	39.47 ± 9.77

Bone weight and bodyweight measures in male and female cohorts. All data given as mean ± S.D. for each group of samples (n ≥ 5 per genotype). Significance was assessed by one way ANOVA with Tukey's post hoc test.

⁎ *p* < 0.05.

⁎⁎ *p* < 0.01.

a Comparison between Wt and *Mecp2*^stop/y^.

b Comparison between *Mecp2*^stop/y^ and *Mecp2*^stop/y^, *CreER*.

**Table 2:** μCT results showing cortical and trabecular bone parameter in Wt, *Mecp2*^stop/y^ and *Mecp2*^stop/y^, *CreER*.

Trabecular bone parameters				Cortical bone parameters			
Trabecular bone parameters	Wild-type	*Mecp2*^stop/y^	*Mecp2*^stop/y^, *CreER*	Cortical bone parameters	Wild-type	*Mecp2*^stop/y^	*Mecp2*^stop/y^, *CreER*
Bone volume fraction%	12.35 ± 5.75	11.13 ± 4.67	16.99 ± 8.92	Outer perimeter (mm)	1.65 ± 0.22	1.32 ± 0.07⁎, a	1.38 ± 0.05⁎, c
Bone mineral density g/cm^3^	0.96 ± 0.07	0.93 ± 0.068	0.94 ± 0.60	Inner perimeter (mm)	1.26 ± 0.08	1.12 ± 0.07⁎, a	1.08 ± 0.05⁎⁎, c
Bone surface density mm^2^/mm^3^	8.15 ± 3.05	8.93 ± 3.11	11.29 ± 5.99	Cortical thickness (mm)	0.41 ± 0.17	0.19 ± 0.07⁎, a	0.21 ± 0.08⁎, c
Specific bone surface mm^2^/mm^3^	70.25 ± 12.73	78.10 ± 17.92	66.14 ± 9.90	Marrow area (mm^2^)	0.48 ± 0.14	0.30 ± 0.02⁎⁎, a	0.29 ± 0.03⁎⁎, c
Connectivity density 1/mm^3^	215.90 ± 93.80	180.90 ± 47.80	271.60 ± 111.25	Cortical area (mm^2^)	0.81 ± 0.08	0.75 ± 0.13	0.69 ± 0.04
Structure model index	1.03 ± 0.56	0.72 ± 0.67	1.00 ± 0.48	Total area (mm^2^)	1.26 ± 0.17	1.05 ± 0.11⁎, a	0.98 ± 1.04⁎⁎, c
Trabecular number 1/mm	1.65 ± 0.52	2.12 ± 0.63	1.81 ± 0.72	Bone volume (mm^3^)	1.75 ± 0.21	1.39 ± 0.19⁎, a	1.39 ± 0.11⁎, c
Trabecular thickness mm	0.07 ± 0.02	0.05 ± 0.02⁎, a	0.09 ± 0.02⁎⁎, b				
Trabecular separation mm	0.62 ± 0.36	0.48 ± 0.26	0.14 ± 0.31				
Degree of anisotropy	2.63 ± 1.31	2.95 ± 0.88	2.46 ± 0.59				
Mean intercept length	0.15 ± 0.04	0.11 ± 0.03	0.32 ± 0.35				

Summary table showing μCT analysis of cortical (tibia midshaft) and trabecular (body of 5th lumbar vertebrae) bone. All data given as mean ± S.D. for each group of samples (n ≥ 5 per genotype). Significance was assessed by one way ANOVA with Tukey's post hoc test.

⁎ *p* < 0.05.

⁎⁎ *p* < 0.01.

a Comparison between Wt and *Mecp2*^stop/y^.

b Comparison between *Mecp2*^stop/y^ and *Mecp2*^stop/y^, *CreER*.

c Comparison between Wt and *Mecp2*^stop/y^, *CreER*.
